# Prognostic Role of MicroRNA-221 in Various Human Malignant Neoplasms: A Meta-Analysis of 20 Related Studies

**DOI:** 10.1371/journal.pone.0087606

**Published:** 2014-01-27

**Authors:** Jie Yang, Jia-yi Zhang, Jing Chen, Yang Xu, Ning-hong Song, Chang-jun Yin

**Affiliations:** 1 Department of Urology, First Affiliated Hospital of Nanjing Medical University, Nanjing, Jiangsu, China; 2 Department of General Surgery, Nanjing First Hospital Affiliated to Nanjing Medical University, Nanjing, Jiangsu, China; University of Pennsylvania School of Medicine, United States of America

## Abstract

**Background:**

MicroRNA-221 (miR-221) has been shown to play an important role in cancer prognosis. In order to evaluate the predictive value of miR-221, we compiled the evidence from 20 eligible studies to perform a meta-analysis.

**Design:**

All of relevant studies were identified by searching PubMed, Embase, and Web of Science, and were assessed by further quality evaluation. Pooled hazard ratios (HRs) with 95% confidence intervals (CIs) of total and stratified analyses, for overall survival (OS) and recurrence-free survival (RFS), were calculated to investigate the association between high miR-221 expression and cancer prognosis.

**Results:**

We found that high miR-221 expression can predict a poor OS in malignant tumors (pooled HR = 1.55, P = 0.017) but has no significant association with RFS (pooled HR = 1.02, P = 0.942). Further in stratified analyses, high miR-221 expression was significantly associated with a poor OS in Asians (pooled HR = 2.04, P = 0.010) or serum/ plasma subgroup (pooled HR = 2.28, P<0.001), and even showed significantly poor OS (pooled HR = 1.80, P<0.001) and RFS (pooled HR = 2.43, P = 0.010) in hepatocellular carcinoma (HCC) subgroup, but was correlated to a favorable RFS in prostate cancer subgroup (pooled HR = 0.51, P = 0.004).

**Conclusions:**

Our findings demonstrate that miR-221 is more suitable to predict cancer prognosis in Asians, and it is a promising prognostic biomarker for HCC. The detection of miR-221 in serum or plasma samples may make it become an effective method for monitoring patients' prognosis and assessing therapeutic efficacy in the future.

## Introduction

Since the discovery of microRNAs (miRNAs) in 1993 [1], emerging studies have suggested that miRNAs are potential regulators of a wide range of biological processes including development, cell differentiation, proliferation, and apoptosis [2]–[8]. MiRNAs are endogenous, small, single-stranded, non-coding RNAs, which negatively regulate gene expression at posttranscriptional level [3]. Aberrant expression of many miRNAs has been discovered in various human carcinomas [9]–[13], so more and more researchers are willing to consider multifarious miRNAs as diagnostic or prognostic biomarkers.

MicroRNA-221 (miR-221), encoded on human chromosome X, is overexpressed in many aggressive carcinomas [14]–[17]. It has been observed that there is an inverse relationship between the expression of miR-221 and some cell cycle inhibitors, such as p27Kip1 [18]–[21]. Abnormal overexpression of miR-221 strongly facilitates tumor cell growth by inducing cell lines in vitro to progress into the S phase of cell cycle [20], [22]. Recently, studies have discovered that miR-221 is significantly up-regulated in cell lines [23]–[26], plasma or serum [27]–[32], and tissues [33]–[39] of numerous human malignancies. Data from clinical studies also indicate that high miR-221 expression is correlated with poor prognosis in glioma [25], [36], [37], breast cancer [15], [16], [40], multiple myeloma [19], [41], hepatocellular carcinoma (HCC) [42]–[44], pancreatic cancer [24], [33], T-cell acute lymphoid leukemia (T-ALL) [35], [45], and gastric cancer [26], [38]. Furthermore, elevated expression of miR-221 in certain carcinomas is obviously related to a trend of easier invasion [15]–[17], [36], [38], [47], [48], larger tumor size [17], [30], [47], earlier metastasis [17], [38], [46] and shorter time to recurrence [30], [39], [48].

However, controversy about the oncogenic role of miR-221 still exists. Some studies draw statistically insignificant conclusions [37], [40], [43], [49]–[51], and even some come to completely opposite results [52]–[54]. Regardless of these inconsistent outcomes, miR-221 is still considered an attractive biomarker for the assessment of cancer survival and recurrence. Therefore, we conducted a meta-analysis to clarify the accurate role of miR-221 for OS and RFS in multiple human malignant neoplasms.

## Materials and Methods

### 1 Search strategy

We conducted an online search using PubMed, Embase and Web of Science for original articles analyzing the prognostic value of miR-221 in various cancers. We selected studies according to varying combinations of the following sets of keywords: ‘cancer’, ‘carcinoma’, ‘neoplasm’, ‘tumour’, ‘tumor’, ‘microRNA-221’, ‘microrna-221’, ‘miRNA-221’, ‘miR-221’, ‘overall survival’, ‘recurrence’, and ‘prognosis’. The last search update was performed on August 28, 2013. All eligible studies published in English were reviewed, and their bibliographies were also examined for other relevant publications. Relevant review articles were manually searched to find additional eligible studies. If more than one article had been published using the same series of study subjects, we only chose the most recent or complete study for this meta-analysis.

### 2 Inclusion and exclusion criteria

**Figure 1:**
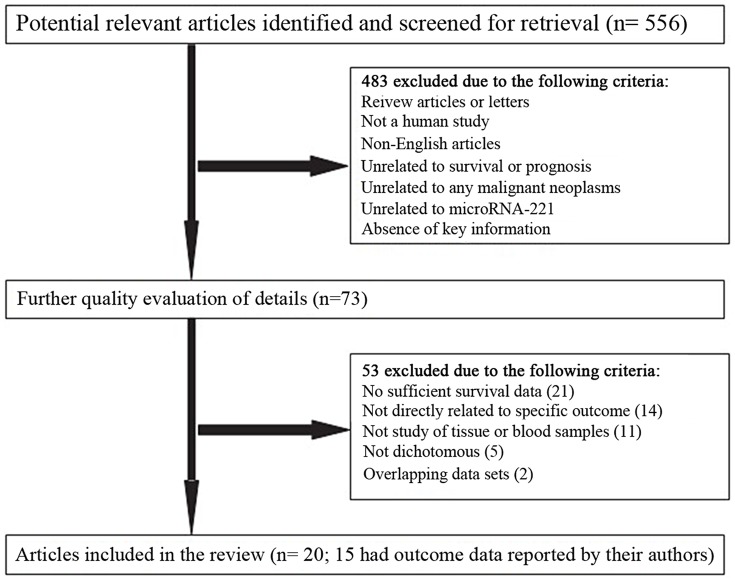
Flow diagram of study selection process.

We followed the guidelines of Preferred Reporting Items for Systematic Reviews and Meta-Analysis (PRISMA) Statement issued in 2009. Articles were considered eligible when they fit the following criteria: (i) MiR-221 was involved in the research; (ii) patients with any malignant tumor were studied; (iii) the relationship between miR-221 expression levels and patients' survival outcomes was investigated. Studies that met above mentioned eligibility criteria were further evaluated and excluded based on a selection process presented in Figure 1.

### 3 Data extraction

All data were carefully extracted, in duplicate, from the eligible publications by two co-authors (J.Y. and J.C.), and any disagreements were resolved by discussion between the two authors. The extracted data elements were exhibited in Table 1 and 2. If HRs and 95% CIs were not reported directly, we extracted the data from Kaplan-Meier curves of survival outcomes to extrapolate required data using the previously described methods [55]–[57]. We also sent e-mails to the corresponding authors of eligible articles requesting additional information and original data needed for the meta-analysis.

**Table 1:** The main characteristics of enrolled studies.

First author & publishing year	Case nationality	Dominantethnicity	Studydesign	Malignant disease	Main type of pathology	Detected sample	Survival analysis	Source of HR	Max months for follow-up
Rong 2013	China	Asian	R	HCC	Adenoca	Tissue	RFS	SC	25
Karakatsanis 2013	Greece	Caucasian	R	HCC	Adenoca	Tissue	OS	DE	84
Gimenes 2013	Brazil	Caucasian	R	T-ALL	Leukemia	Tissue	OS	Reported	140
Hong 2013	China	Asian	R	Ovarian cancer	Adenoca	Serum	OS	Reported	84
Aysegül 2013	Austria	Caucasian	R	Brain glioma	Glioma	Tissue	RFS	DE	47
Amankwah 2013	USA	Caucasian	R	Prostate cancer	Adenoca	Tissue	RFS	Reported	254
Zhang 2012	China	Asian	R	Brain glioma	Glioma	Tissue	OS	SC	48
Liu 2012	China	Asian	R	Gastric cancer	Adenoca	Tissue	OS	Reported	70
Kang 2012	Korea	Asian	R	Prostate cancer	Adenoca	Tissue	RFS	Reported	55
Hanna 2012	USA	Caucasian	R	Breast cancer	Adenoca	Tissue	OS	Reported	250
Yoon 2011	Korea	Asian	R	HCC	Adenoca	Tissue	RFS	Reported	54
Radojicic 2011	Greece	Caucasian	R	Breast cancer	Adenoca	Tissue	OS	SC	120
Li 2011	China	Asian	R	HCC	Adenoca	Serum	OS	Reported	60
Spahn 2010	Germany	Caucasian	R	Prostate cancer	Adenoca	Tissue	RFS	Reported	111
Duncavage 2010	USA	Caucasian	R	NSCLC	Adenoca, SqCa	Tissue	RFS	SC	60
Wurz 2010	USA	Caucasian	R	Ovarian cancer	Adenoca	Tissue	OS	Reported	60
Wang 2010	China	Asian	R	AML/ ALL	Leukemia	Tissue	OS	Reported	33
Pu 2010	China	Asian	R	Colorectal cancer	Adenoca	Plasma	OS	Reported	60
Guo 2010	China	Asian	R	Lymphoma	Lymphoma	Plasma	OS	Reported	60
Gramantieri 2009	Italy	Caucasian	R	HCC	Adenoca	Tissue	OS, RFS	SC	133

Study design is described as consecutive patients (C), prospective (P) or retrospective (R).

HCC, hepatocellular carcinoma; T-ALL, T-cell acute lymphoid leukemia; AML, acute myeloid leukemia; NSCLC, nonsmall-cell lung cancer; Adenoca, adenocarcinoma; SqCa, squamous carcinoma; OS, overall survival; RFS, recurrence-free survival; HR, hazard ratio; SC, survival curve; DE, data-extrapolated.

**Table 2:** The difference of overall survival and recurrence-free survival between high- and low-expression cases of microRNA-221 in enrolled studies.

First author & publishing year	Assay method	Cut-off value	Case number		OS		RFS	
			high expression	low expression	HR (95% CI)	P	HR (95% CI)	P
Rong 2013	qRT-PCR	Median	24	24	NM	NM	1.60 (0.88, 2.90)^U^*	0.129
Karakatsanis 2013	qRT-PCR	Mean	NM	NM	1.79 (1.50, 2.13)^M,DE^	0.000	NM	NM
Gimenes 2013	qRT-PCR	Median	24	24	2.31 (0.92, 5.81)^M^	0.074	NM	NM
Hong 2013	qRT-PCR	Mean	51	45	2.24 (1.13, 4.48)^M^	0.020	NM	NM
Aysegül 2013	qRT-PCR	Median	5	10	NM	NM	1.11 (0.37, 3.35)^DE^	0.855
Amankwah 2013	qRT-PCR	Median	28	35	NM	NM	0.56 (0.21, 1.50)^M^	0.250
Zhang 2012	qRT-PCR	Mean	22	14	2.62 (1.19, 5.75)^U^*	0.011	NM	NM
Liu 2012	qRT-PCR	Mean	48	44	2.32 (1.11, 4.85)^M, DE^	0.025	NM	NM
Kang 2012	qRT-PCR	Median	NM	NM	NM	NM	0.36 (0.17, 1.90)^U^	0.570
Hanna 2012	qRT-PCR	Highest tertile	354	119	0.70 (0.51, 0.97)^M^	0.312	NM	NM
Yoon 2011	qRT-PCR	Mean	30	85	NM	NM	2.09 (1.09, 4.04)^M^	0.027
Radojicic 2011	qRT-PCR	Median	49	38	1.62 (0.76, 3.47)^U^*	0.458	NM	NM
Li 2011	qRT-PCR	4.8-fold	21	25	1.90 (1.24, 2.98)^U^	<0.05	NM	NM
Spahn 2010	qRT-PCR	Median	49	43	NM	NM	0.53 (0.29, 0.95)^M^	0.032
Duncavage 2010	qRT-PCR	Mean	20	21	NM	NM	0.41 (0.14, 1.15)^U^*	0.120
Wurz 2010	qRT-PCR	Median	NM	NM	0.32 (0.13, 0.82)^U^	0.010	NM	NM
Wang 2010	qRT-PCR	Median	16	16	0.54 (0.30, 0.97)^U, DE^	0.038	NM	NM
Pu 2010	qRT-PCR	Mean	19	80	3.48 (1.04, 11.65)^M^	0.043	NM	NM
Guo 2010	qRT-PCR	Mean	50	29	5.71 (1.78, 18.18)^M^	0.003	NM	NM
Gramantieri 2009	qRT-PCR	Median	21	25	1.64 (0.67, 4.05)^U^*	0.500	6.60 (2.15, 20.21)^U^*	0.001

qRT-PCR, quantitative real-time PCR; NM, not mentioned; OS, overall survival; CI, confidence interval; RFS, recurrence-free survival; HR, hazard ratio; *, HR and 95% CI calculated by survival curve; M, cox multivariate analysis; U, cox univariate analysis; DE, data-extrapolated.

### 4 Statistical analysis

All statistical analyses were conducted using Stata®11 (StataCorp LP, College Station, TX, USA) and Microsoft Excel (Version 2007, Microsoft corp., Redmond, WA, USA). The aggregation of HRs and 95% CIs were calculated following Tierney's method [57]. Forest plots were used to estimate the effect of miR-221 expression on survival outcome (OS and RFS). The heterogeneity assumption of pooled HRs was verified by Cochran's Q-test, and the percentage of Higgins I-squared statistic (I^2^) was used to quantify the extent of heterogeneity explained by the characteristics of enrolled studies. If significant heterogeneity was observed (P<0.1 or I^2^>50%), a random-effects model (DerSimonian-Laird method) was applied, otherwise the fixed-effects model (Mantel-Haenszel method) was used [58]. To avoid the influence of heterogeneity, we also conducted subgroup analyses based on similar characteristics, such as dominant ethnicity, main type of pathology, and detected sample category. Potential publication bias was determined by Egger's linear regression test with a funnel plot [59]. All P values were two-sided and a P value of less than 0.05 was considered to be statistically significant.

## Results

### 1 Summary of included studies

According to the study selection process, 556 studies on miR-221 and cancer were identified from a primary literature search in Pubmed, Embase, and Web of Science. Four hundred eighty-three studies were excluded based on manual screening of the title and the abstract, and 53 were further removed by assessment on the full text (Figure 1). Finally, 20 studies, which investigated the potential relationship between miR-221 expression and patients' survival or disease recurrence in various malignant neoplasms, were considered eligible for this meta-analysis.

Analyzed data of enrolled studies were collected from the United States, Germany, Greece, Italy, Austria, China, Korea, and Brazil. The dominant ethnicity was Caucasian in half of the enrolled studies, while the other 10 studies were executed in Asians. All of the studies were retrospective in design, and the maximum follow-up was from 25 to 254 months. The malignant diseases involved in this review included HCC, acute leukemia, ovarian cancer, glioma, prostate cancer (PCa), gastric cancer, breast cancer, non-small cell lung cancer (NSCLC), colorectal cancer (CRC), and lymphoma. The expression level of miR-221 was usually detected by quantitative real-time polymerase chain reaction (qRT-PCR) assay in tissue samples, while four studies tested it in plasma or serum samples. (Table 1)

Among these studies, 12 focused on OS, seven were associated with RFS, and one evaluated both OS and RFS. Thirteen studies directly reported HRs and 95% CIs. We calculated these necessary statistical variables by survival curves in five studies, and extrapolated them based on available numerical data in the other two studies. (Table 1)

### 2 High miR-221 expression and overall survival

**Figure 2:**
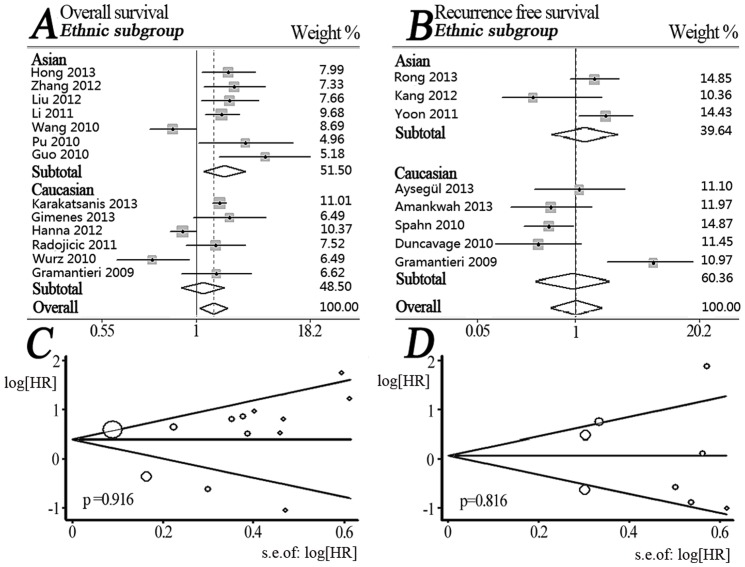
Forest plots for merged analyses of overall survival (OS) and recurrence-free survival (RFS) associated with miR-221 expression difference, and Begg's funnel plots of publication bias test. (A) Forest plots of overall and ethnic subtotal analyses of OS. Squares and horizontal lines correspond to study-specific HRs and 95% CIs, respectively. The area of the squares correlates the weight of each enrolled study and the diamonds represent the summary HRs and 95% CIs; (B) Forest plots for overall and ethnic subtotal analyses of RFS; (C) Begg's funnel plots of publication bias test for the overall merged analysis of OS. Each point represents a separate study; (D) Begg's funnel plots of the publication bias test for the overall merged analysis of RFS.

A total of 13 articles were involved in OS analysis (Table 2, Figure 2A), among which significant heterogeneity was observed (P = 0.000, I^2^ = 80.5%). Hence, a random model was applied to calculate a pooled HR and 95% CI, and we found that patients with high miR-221 expression had a significantly poorer OS when compared to individuals with a low expression of miR-221 (HR = 1.55, 95% CI, 1.08–2.22) (Table 3).

**Table 3:** The pooled HRs, 95% CIs and p values of overall survival and recurrence-free survival stratified by ethnicity, main pathologic type, categories of malignant diseases and detected samples.

Subgroup	Overall survival			Recurrence free survival		
	N	HR (95% CI)	p value	N	HR (95% CI)	p value
Total	13	1.55 (1.08, 2.22)^b^	0.017	8	1.02 (0.55, 1.89)^b^	0.942
*Ethnic subtotal*						
Caucasian	6	1.17 (0.67, 2.03)^b^	0.578	5	0.94 (0.39, 2.27)^b^	0.883
Asian	7	2.04 (1.19, 3.49)^b^	0.010	3	1.25 (0.56, 2.77)^b^	0.586
*Main pathologic subtotal*						
Adenocarcinoma	9	1.46 (0.98, 2.18)^b^	0.062	6	1.16 (0.55, 2.42)^b^	0.698
Leukemia or lymphoma	3	1.80 (0.44, 7.41)^b^	0.415	-	-	-
*Malignant disease subtotal*						
Hepatocellular carcinoma	3	1.80 (1.53, 2.11)^a^	<0.001	3	2.43 (1.24, 4.77)^b^	0.010
Ovarian cancer	2	0.87 (0.13, 5.84)^b^	0.884	-	-	-
Breast cancer	2	0.99 (0.44, 2.22)^b^	0.980	-	-	-
Prostate cancer	-	-	-	3	0.51 (0.32, 0.81)^a^	0.004
*Detected sample subtotal*						
Tissue	9	1.25 (0.80, 1.95)^b^	0.336	8	1.02 (0.55, 1.89)^b^	0.942
Serum or plasma	4	2.28 (1.62, 3.19)^a^	<0.001	-	-	-

N, number of studies; HR, hazard ratio; CI, confidence interval.

a, the HRs and 95% CIs of enrolled studies are pooled by the fixed-effects model; b, the HRs and 95% CIs of enrolled studies are pooled by the random-effects model if p value for heterogeneity test was less than 0.10 or I^2^ was more than 50%.

**Figure 3:**
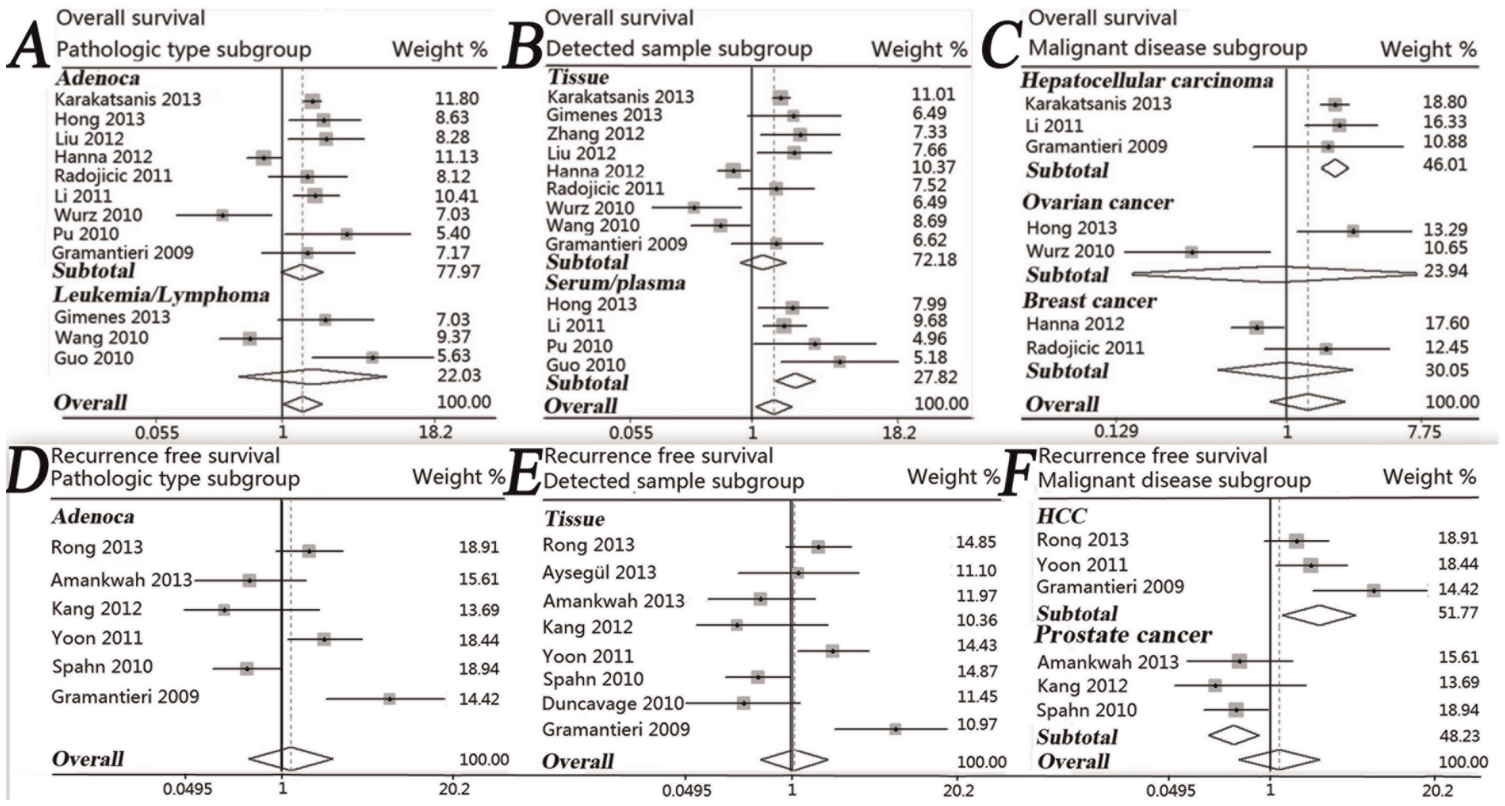
Forest plots for merged analyses of overall survival (OS) and recurrence-free survival (RFS), associated with miR-221 expression difference, in different subgroups. (A) Forest plots for the subgroup analysis of OS in different pathological types. Squares and horizontal lines correspond to study-specific HRs and 95% CIs, respectively. The area of the squares correlates the weight of each enrolled study and the diamonds represent the summary HRs and 95% CIs; (B) Forest plots for the subgroup analysis of OS in different detected samples; (C) Forest plots for the subgroup analysis of OS in different malignant diseases; (D) Forest plots for the subgroup analysis of RFS in adenocarcinoma; (E) Forest plots for the subgroup analysis of RFS in tissue samples; (F) Forest plots for the subgroup analysis of RFS in different malignant diseases.

In order to avoid the influence of heterogeneity, we conducted four subtotal analyses stratified by dominant ethnicity, main pathologic type, categories of detected samples, and malignant diseases. First, seven studies in Asians [29]–[32], [36], [38], [52] showed that increased expression of miR-221 predicted a significantly worse OS (HR = 2.04, 95% CI: 1.19–3.49) by a random-effects model due to significant heterogeneity among pooled studies (P = 0.001, I^2^ = 73.7%). We didn’t find a significantly worse OS in Caucasians with high miR-221 expression by merging six studies [35], [40], [44], [47], [51], [53] (Figure 2A). In subtotal analyses of main pathologic type category, no statistically significant result was observed in adenocarcinoma and leukemia/lymphoma subgroup (Figure 3A). When stratified by the category of detected samples, increased expression of miR-221 showed a significant association with poor OS (HR = 2.28, 95% CI: 1.62–3.19) by a fixed-effects model (P = 0.316, I^2^ = 15.2%) in serum/plasma subgroup [29]–[32], but no significant relationship was observed in tissue subgroup (Figure 3B). Moreover, in subtotal analysis stratified by the category of malignant diseases, three studies of HCC [30], [44], [47] exhibited a significant association between increased expression of miR-221 and poor OS (HR = 1.80, 95% CI: 1.53–2.11) by a fixed-effects model (P = 0.950, I^2^ = 0.0%). However, we did not discover any significant association in subgroups of ovarian cancer or breast cancer (Figure 3C).

### 3 High miR-221 expression and recurrence-free survival

A total of eight studies focused on RFS analysis (Table 2, Figure 2B) with a significant heterogeneity among them (P = 0.000, I^2^ = 76.1%). A random-effects model was applied for merging overall data, but no obvious relationship between increased expression of miR-221 and RFS was observed (HR = 1.02, 95% CI: 0.55–1.89) (Table 3).

Similar to OS analyses, we also performed subtotal investigation for RFS analyses. When stratified by dominant ethnicity, no significant association was observed in both Caucasians and Asians (Figure 2B). In subtotal analyses of main pathological type, the pooled outcome of adenocarcinoma subgroup did not reveal high miR-221 expression could significantly predict a poor RFS (Figure 3D). When stratified by the category of detected samples, the outcome of tissue subgroup also didn’t show statistical significance (Figure 3E). Finally, stratified by the category of malignant diseases, elevated expression of miR-221 exhibited a significant association with poor RFS (HR = 2.43, 95% CI: 1.24–4.77) in HCC [39], [43], [47] by a random-effects model (P = 0.091, I^2^ = 58.3%). However, the pooled outcome in PCa subgroup [48], [49], [54] surprisingly showed elevated miR-221 expression was significantly associated with a favorable RFS (HR = 0.51, 95% CI: 0.32–0.81) by a fixed-effects model (P = 0.831; I^2^ = 0.0%) (Figure 3F).

### 4 Publication bias

Publication bias, for total OS or RFS analyses, was respectively evaluated by funnel plots. The shape of all funnel plots seemed symmetrical suggesting absence of a publication bias (Figure 2C and 2D). Egger's test was used to provide statistical evidence for funnel plot symmetry. As expected, the P value of Egger's test was 0.916 for OS and 0.816 for RFS. Hence, there was no evidence for significant publication bias in the meta-analysis.

## Discussion

Compared to mRNAs, miRNAs are more stable and not easily degraded. They exhibit a special expression profile in various normal and malignant tissues, which can be accurately detected and quantified by qRT-PCR [60] not only in frozen or fresh tissues, but also in formalin-fixed paraffin-embedded tissues. They can also be quantified in serum or plasma samples, and even in urine or saliva samples [61]. In recent studies, miR-221 has been found closely associated with tumors by intricate regulatory mechanisms, in which several target genes of miR-221 affect tumorigenesis and progression. For instance, miR-221 can suppress the expression of cell cycle regulators, p27Kip1 and p57Kip2 mRNA, in multiple cancers to induce the proliferation of tumor cells [18]–[21]. MiR-221 also blocks the migration and proliferation of tumor cells and the angiogenesis of tumor tissues by targeting c-kit, the stem cell factor receptor [62]. Moreover, increased expression of miR-221 is able to inhibit cell apoptosis in HCC by negatively regulating Bcl2 modifying factor (BMF), a well-known factor involved in the balance between proapoptosis and antiapoptosis [47]. Pu et al find that the expression of p53, a tumor suppressor, is negatively correlated with the plasma level of miR-221, and suggest that p53 might repress miR-221 expression in CRC [31]. Zhang et al confirm that miR-221 directly inhibits the posttranscriptional expression of metallopeptidase inhibitor 3 (TIMP3), an inhibitor of matrix metalloproteinases (MMPs), and plays an important role in promoting the invasion of human gliomas [36]. The oncogenic effect of miR-221 is also mediated by phosphatase and tensin homolog (PTEN) [38]. Currently, a number of clinical studies have shown a significant correlation between the expression level of miR-221 and the prognosis of various malignant tumors [29]-[32]. However, the results are not consistent and even contradictory, which may be due to the differences in disease categories, ethnic affiliations, and detected samples. Therefore, it is necessary to conduct stratified pooled analyses to identify the prognostic value of miR-221 in survival and recurrence, as well as its application scope.

By stratified analyses of enrolled studies, we successfully drew some valuable conclusions for clinical application. First, in order to exclude the interference caused by the different genetic backgrounds of patients, the 20 enrolled studies were classified based on ethnic affiliation into Asians and Caucasians (Table 1). We found increased miR-221 expression predicts a significantly worse OS in Asians (pooled HR = 2.04, P = 0.010), but there was no statistical significance in Caucasians (pooled HR = 1.17, P = 0.578). This observation may be due to the difference in hereditary backgrounds and environmental exposures. Plenty of researches have also shown different expression levels and predictive values of miRNAs in various ethnic groups [63]–[65]. Therefore, we consider that diverse hereditary backgrounds and environmental exposures give rise to different predictive values of miR-221 in cancer prognosis, and miR-221 is more suitable as a tumor biomarker for prognosis in Asians.

Second, we performed subgroup analyses on the basis of pathological types. Due to the limited number of eligible studies, only two subgroups of adenocarcinoma and leukemia/lymphoma could be further analyzed, however, we failed to find any statistically significant results in the two subgroups (Table 3, Figure 3). Hence the type of tumors, in which miR-221 can be suitably utilized as a prognostic marker, still needs to be determined.

To further exclude the differences of tumorigenesis and development mechanisms among various cancers, we classified the enrolled studies into subgroups of cancer categories. It was observed that high miR-221 expression is significantly associated with both poor OS (pooled HR = 1.80, P<0.001) and RFS (pooled HR = 2.43, P = 0.010) in HCC (Table 3, Figure 3). HCC patients with high miR-221 expression exhibit a significantly decreased survival rate and a significantly increased recurrence rate than those with low expression of miR-221. It may be because that elevated miR-221 may be able to induce tumor cell proliferation by negatively regulating the expression of p27Kip1 and p57Kip2, as well as inhibit cell apoptosis by suppressing the expression of BMF [47]. Hence, we consider high miR-221 expression as a promising risk biomarker for poor prognosis in HCC. However, we draw a completely opposite conclusion in PCa subgroup [48], [49], [54] where high miR-221 expression predicts a significantly lower recurrence risk (pooled HR = 0.51, P = 0.004). Spahn et al find that miR-221 is commonly down-regulated in PCa, which has no relation with the mRNA levels of p27Kip1, but significantly correlates to the overexpression of c-kit [54]. Researchers, therefore, consider c-kit to play a key role in promoting tumorigenesis [54] and bone metastasis of PCa [66].

Finally, in order to clarify the prognostic values of miR-221 expression level in different clinical samples, we classified the enrolled studies into subgroups of tissue samples and serum/plasma samples. We found that high expression of miR-221 significantly relates to a poor OS (pooled HR = 2.28, P<0.001) in serum/plasma subgroup. No statistical significance is shown in tissue subgroup (pooled HR = 1.25, P = 0.336) (Table 3, Figure 3). Although detection of miR-221 in tissue samples is widely used in current research for tumor prognosis, detection by serum/ plasma samples is more convenient and faster, which can effectively evaluate both survival prognosis and recurrence risk at any time point during or after clinical therapy, and even can keep monitored through the lifetime of patients. Therefore, detection of serum/ plasma miR-221 during follow-up may be an efficacious method for dynamically monitoring the prognosis and therapeutic effects in cancer patients.

These results indicate that miR-221 can be used for predicting cancer prognosis, and it is a promising biomarker. However, some details need to be further refined. First, there are only 20 eligible articles included in our analyses, which leads to the relative insufficiency of studies in subgroup analyses. When there are less than two studies for certain cancer prognoses, subgroup analysis cannot be carried out. Besides, there is no independent study in Africans for the meta-analysis, which hinders the comprehensive investigation of the association between miR-221 expression and cancer prognosis. Second, due to the lack of uniform cut-off value in miR-221 expression, different researchers apply different cut-off values, which may be higher or lower than the actual value and would affect the effectiveness of miR-221 as a predictive factor in cancer prognosis. Third, the pooled value of HR for total OS analysis is 1.55 in patients with high miR-221 expression, which is statistically significant (P = 0.017) but not strong enough. Empirically, a predictive factor is considered to be strong when the value of HR is more than 2.0 [67]. Fourth, although the pooled outcome of three studies shows increased expression of miR-221 is significantly associated with a favorable RFS in PCa (pooled HR = 0.51, P = 0.004), two of the three studies show no statistical significance (Table 2, Figure 3). Therefore, the value of miR-221 as a prognostic marker for PCa is still arguable, requiring more research for confirmation. Fifth, it still needs to be verified if miR-221 can be used as an independent tumor biomarker, or if miR-221 should be part of a combination of biomarkers utilized for predicting tumor prognosis. Using Cox proportional hazards regression analysis, Wang, et al evaluated a linear combination of the expression values of three miRNAs (miR-146a, miR-181a/c, and miR-221), and found that the combined value exhibited an obvious negative correlation to the OS of ALL patients (r = −0.5933, P = 0.0039). However when analyzed separately, high expression of miR-146a (HR = 1.69, P = 0.039) and miR-181a/c (HR = 1.70,P = 0.011) both indicate a significantly poor prognosis, whereas high miR-221 expression is associated with a favorable prognosis (HR = 0.54, P = 0.038). In addition several drawbacks, such as a relatively small number of enrolled patients [37] or a short follow-up time [43], exist in individual studies. Therefore, further research on miR-221 for predicting cancer prognosis is required to confirm the prognostic role of miR-221.

Our meta-analysis also has some advantages. First, we strictly followed the literature inclusion criteria and the quality of enrolled literatures was satisfactory. Second, we conducted a multi-stratified analysis to effectively minimize the influence of heterogeneity among the enrolled studies, and to further explore the scope of application for miR-221 as a prognostic biomarker of malignant tumors. Third, no significant publication bias is found in our meta-analysis (Figure 2). All these advantages have increased the statistical power of the meta-analysis.

## Conclusions

In summary, we conclude that miR-221 is suitable to predict tumor prognosis in Asian populations, and is an ideal prognostic biomarker for HCC patients. Besides, detecting miR-221 expression in serum/ plasma samples is more convincing to predict a poor prognosis than detection of miR-221 in tissue samples. Detection of miR-221 in human peripheral blood samples possesses the advantages of low cost, convenience, and non-invasion, resulting in an effective method in monitoring cancer progression as well as assessing therapeutic efficacy in future. Considering the paucity of relevant data, further investigation and more studies are needed to focus on the relationship between the expression of miR-221 and cancer prognosis.

## Supporting Information

Checklist S1 PRISMA checklist.
(DOC)

File S1 PRISMA flow diagram.
(DOC)
