# Supplementary material for: Prognostic Role of MicroRNA-221 in Various Human Malignant Neoplasms: A Meta-Analysis of 20 Related Studies
Source: PLoS One. 2014 Jan 27;9(1):e87606. doi: 10.1371/journal.pone.0087606 (PMC3903772; doi:10.1371/journal.pone.0087606)
Supplement: Checklist S1 — PRISMA checklist. (DOC) [file pone.0087606.s001.doc]

| **Section/topic** | **#** | **Checklist item** | **Reported on page #** |
| --- | --- | --- | --- |
| **TITLE** | | |  |
| Title | 1 | Prognostic role of microRNA-221 in various human malignant neoplasms: a meta-analysis of 20 related studies | Article title |
| **ABSTRACT** | | |  |
| Structured summary | 2 | Background: MicroRNA-221 (miR-221) has been shown to play an important role in cancer prognosis. In order to evaluate the predictive value of miR-221, we compiled evidence from 20 eligible studies to perform a meta-analysis.  Design: The 20 relevant studies were identified by searching PubMed, Embase, and Web of Science. The studies were further assessed by quality evaluation of the data. To investigate the association between elevated miR-221 and cancer prognosis, the pooled hazard ratios (HRs) with 95% confidence intervals (CIs) of total and stratified analyses, for overall survival (OS) and recurrence-free survival (RFS), were calculated.  Results: We found that increased expression of miR-221 can predict a poor OS in malignant tumors (pooled HR = 1.55, P = 0.017) but has no significant association with RFS (pooled HR = 1.02, P = 0.942). Further in stratified analyses, elevated miR-221 was significantly associated with worse OS in the Asian subgroup (pooled HR = 2.04, P = 0.010) and serum/ plasma subgroup (pooled HR = 2.28, P < 0.001). Elevated miR-221 showed both, worse OS (pooled HR = 1.80, P < 0.001) and worse RFS (pooled HR = 2.43, P = 0.010), in the hepatocellular carcinoma (HCC) subgroup. However, a similar elevated expression of miR-221 was inversely correlated to a better RFS in prostate cancer (pooled HR = 0.51, P = 0.004).  Conclusions: Our findings demonstrate that miR-221 is an appropriate biomarker of cancer prognosis in Asians, as well as a promising prognostic biomarker for HCC. Besides, detection of miR-221 in serum or plasma samples would become an effective method in monitoring cancer progression, and in assessing therapeutic strategies in the future.  Keywords: malignant tumor, miR-221, prognosis, OS, RFS | Abstract |
| **INTRODUCTION** | | |  |
| Rationale | 3 | Since the discovery of microRNAs (miRNAs) in 1993, emerging studies have suggested miRNAs are potential regulators of a wide range of biological processes that include development, cell differentiation, proliferation, and apoptosis. MicroRNAs are endogenous, small, single-stranded, non-coding RNAs, which negatively regulate gene expression at the posttranscriptional level. Aberrant expression of many miRNAs has been discovered in various human carcinomas, so more and more researchers are willing to consider multifarious miRNAs as diagnostic or prognostic biomarkers.  MicroRNA-221, encoded on human chromosome X, is overexpressed in many aggressive carcinomas. It has been observed that there is an inverse relationship between the expression of miR-221 and some cell cycle inhibitors, such as p27Kip1. Abnormal overexpression of miR-221 strongly facilitates tumor cell growth, by inducing cell lines in vitro to progress to S phase of the cell cycle. Recently, studies have discovered that miR-221 is significantly upregulated in cell lines, plasma or serum, and tissues of numerous human malignancies. Data from clinical research studies also indicate that elevated expression of miR-221 is correlated with poor prognosis in glioma, breast cancer, multiple myeloma, HCC, pancreatic cancer, T-cell acute lymphoid leukemia (T-ALL), and gastric cancer. Furthermore, elevated expression of miR-221 in certain carcinomas facilitates invasion, large tumor size, earlier metastasis, and shorter time to recurrence.  However, controversy about the oncogenic role of miR-221 still exists. While the outcomes of certain studies are statistically insignificant, results of other studies are found to be completely opposed. Regardless of these inconsistent outcomes, miR-221 is still considered an attractive biomarker for the assessment of cancer survival and recurrence. | Introduction |
| Objectives | 4 | We conducted a meta-analysis to clarify the accurate role of miR-221 for OS and RFS in multiple human malignant neoplasms. All studies were retrospective and all patients were divided into high miR-221 expression group and low miR-221 expression group. Finally, pooled outcomes demonstrated that elevated miR-221 expression was significantly associated with poor prognoses. | Introduction |
| **METHODS** | | |  |
| Protocol and registration | 5 | Indicate if a review protocol exists, if and where it can be accessed (e.g., Web address), and, if available, provide registration information including registration number. | n/a |
| Eligibility criteria | 6 | We followed the guidelines of PRISMA (Preferred Reporting Items for Systematic Reviews and Meta-Analysis) Statement issued in 2009. Articles were considered eligible when they fit the following criteria: (i) MicroRNA-221 was involved in the research. (ii) Patients with different types of carcinomas were studied. (iii) The relationship between expression levels of miR-221 and survival outcome of patients was investigated. Studies that fit the eligibility criteria were further evaluated and excluded based on a selection process presented in Figure 1. | Materials and Methods |
| Information sources | 7 | We conducted an online search, in PubMed, Embase, and Web of Science, for original articles analyzing the prognostic value of miR-221 in various cancers. The last search update was performed on August 28, 2013. | Materials and Methods |
| Search | 8 | We selected studies based on varying combinations of the following sets of keywords: ‘cancer’, ‘carcinoma’, ‘neoplasm’, ‘tumour’, ‘tumor’, ‘microRNA-221’, ‘microrna-221’, ‘miRNA-221’, ‘miR-221’, ‘overall survival’, ‘recurrence’, and ‘prognosis’. All eligible studies published in English were reviewed, and their bibliographies were also examined for other relevant publications. Relevant review articles were manually searched to find additional eligible studies. If more than one article had been published using the same series of study subjects, we only chose the most recent or complete study for this meta-analysis. | Materials and Methods |
| Study selection | 9 | State the process for selecting studies (i.e., screening, eligibility, included in systematic review, and, if applicable, included in the meta-analysis). | Figure 1 |
| Data collection process | 10 | All data were carefully extracted, in duplicate, from the eligible publications by 2 coreviewers (J.Y. and J.C.). Disagreements, if any, were resolved by discussion between the two authors. If HR and 95% CI were not reported directly, we extracted the data from Kaplan–Meier curves of survival outcomes to extrapolate the HR and 95% CI using statistical methods described below. We also sent e-mails to the corresponding authors of eligible articles requesting additional information and original data needed for the meta-analysis. | Materials and Methods |
| Data items | 11 | The extracted data elements in Table 1 included the following: the first author’s last name and publishing year, case nationality, dominant ethnicity, study design, malignant disease, main type of pathology, detected sample, category of survival analysis, source of hazard ratio (HR), and maximum months for follow-up. The extracted data elements in Table 2 included assay method, cut off value, case numbers of high and low expression groups, HR, 95% CI, and P value for OS and RFS. | Materials and Methods |
| Risk of bias in individual studies | 12 | Describe methods used for assessing risk of bias of individual studies (including specification of whether this was done at the study or outcome level), and how this information is to be used in any data synthesis. | n/a |
| Summary measures | 13 | Forest plots were used to estimate the effect of miR-221 expression on survival outcome (OS and RFS). All P values were 2-tailed and a P value of less than 0.05 was considered to be statistically significant. | Materials and Methods |
| Synthesis of results | 14 | Forest plots were used to estimate the effect of miR-221 expression on survival outcome (OS and RFS). The heterogeneity assumption of pooled HRs was verified by Cochran Q-test. The percentage of Higgins I-squared statistic (I2) was used to quantify the extent of heterogeneity observed in the characteristics of enrolled studies. If significant heterogeneity was observed (P < 0.1 or I2 > 50%), a random-effects model (DerSimonian and Laird method) was applied, otherwise the fixed-effects model (Mantel-Haenszel test) was used. | Materials and Methods |

Page 1 of 2

| **Section/topic** | **#** | **Checklist item** | **Reported on page #** |
| --- | --- | --- | --- |
| Risk of bias across studies | 15 | Potential publication bias was determined by Egger’s linear regression test with a funnel plot | Materials and Methods |
| Additional analyses | 16 | To avoid the influence of heterogeneity, we also conducted subgroup analyses based on similar characteristics, such as dominant ethnicity, main type of pathology, and detected sample category. | Materials and Methods |
| **RESULTS** | | |  |
| Study selection | 17 | According to the study selection process, 556 studies on miR-221 and cancer were identified from a primary literature search in Pubmed, Embase, and Web of Science. Four hundred eighty-three studies were excluded based on manual screening of the title and abstract, and 53 were further removed by assessment of the full text (Figure 1). Finally, 20 studies, which investigated the potential relationship between the expression of miR-221 and survival of patients or disease recurrence in various malignant neoplasms, were considered eligible for this meta-analysis (Table 1). | Results |
| Study characteristics | 18 | Data analyzed from the enrolled studies were collected from the United States, Germany, Greece, Italy, Austria, China, Korea, and Brazil. The dominant ethnicity was Caucasian in half of the enrolled studies, while the other 10 studies were executed in Asians (Table 1). All the studies were retrospective in design, and the maximum follow-up was from 25 to 254 months. The malignant diseases involved in this review included HCC, acute leukemia, ovarian cancer, glioma, prostate cancer, gastric cancer, breast cancer, non-small cell lung cancer (NSCLC), colorectal cancer (CRC), and lymphoma (Table 1). The expression level of miR-221 was usually detected by quantitative real-time polymerase chain reaction (qRT-PCR) assay in tissue samples, while 4 studies tested levels of miR-221 in plasma or serum samples (Table 1).  Among these studies, 12 focused on OS, 7 were associated with RFS, and 1 evaluated both, OS and RFS (Table 1). Thirteen studies directly reported HRs and 95% CIs, and we calculated the necessary statistical variables for meta-analysis by the Kaplan-Meier survival curves in 5 studies. We extrapolated HR and CI based on available numerical data in the other 2 studies (Table 1). The cut off values of dichotomous expression levels were different in each study, with 10 studies calculating the median expression value for miR-221 and 8 studies calculating the mean expression value. In 1 study, average fold-change of miR-221 expression was used as a threshold, while in a second study, the highest tertile value of miR-221 expression levels was used (Table 2). | Results |
| Risk of bias within studies | 19 | Present data on risk of bias of each study and, if available, any outcome level assessment (see item 12). | n/a |
| Results of individual studies | 20 | For all outcomes considered (benefits or harms), present, for each study: (a) simple summary data for each intervention group (b) effect estimates and confidence intervals, ideally with a forest plot. | Table 2 |
| Synthesis of results | 21 | A total of 13 articles were involved in OS analysis (Table 2, Figure 2A), among which significant heterogeneity was observed (P = 0.000, I2 = 80.5%). Hence, a random model was applied to calculate a pooled HR and 95% CI, and we found that patients with a high expression of miR-221 had a significantly poorer OS when compared to individuals with a lower expression of miR-221 when overall data was merged (HR = 1.55, 95% CI, 1.08-2.22) (Table 3).  A total of 8 studies focused on RFS analysis (Table 2) (Figure 2B), with a significant heterogeneity among them (P = 0.000, I2 = 76.1%). A random-effects model was applied for the merging of overall data, but no obvious relationship between increased expression of miR-221 and RFS (HR = 1.02, 95% CI, 0.55-1.89) was observed (Table 3). | Results |
| Risk of bias across studies | 22 | Publication bias, for total OS or RFS analyses, was respectively evaluated by funnel plots. The shape of all funnel plots seemed symmetrical suggesting absence of a publication bias (Figure 2C and Figure 2D). Egger’s test was used to provide statistical evidence for funnel plot symmetry. As expected, the P value of Egger’s test was 0.916 for OS and 0.816 for RFS. Hence, there was no evidence for significant publication bias in the meta-analysis. | Results |
| Additional analysis | 23 | In order to avoid the influence of heterogeneity, we conducted 4 subtotal analyses stratified by dominant ethnicity, main pathologic type, categories of detected samples, and malignant diseases (Table 3). First, 7 studies in Asians showed that increased expression of miR-221 predicted a significantly worse OS (HR = 2.04, 95% CI, 1.19-3.49) by a random-effects model due to the significant heterogeneity among pooled studies (P = 0.001, I2=73.7%). We didn’t find a poor OS in Caucasians with increased expression of miR-221 by merging 6 studies (Figure 2A). In subtotal analyses of the main pathologic type category, no statistically significant result was observed in the adenocarcinoma subgroup and the leukemia/lymphoma subgroup (Figure 3A). When stratified by the category of detected samples in the serum/plasma subgroup, increased expression of miR-221 showed a significant association with poor OS (HR = 2.28, 95% CI, 1.62-3.19) by a fixed-effects model (P = 0.316, I2 = 15.2%), but no significant relationship between elevated miR-221 levels and poor OS was observed in the malignant tissue subgroup (Figure 3B). Moreover, in subtotal analysis stratified by the category of malignant diseases, 3 studies of HCC exhibited a significant association between increased expression of miR-221 and poor OS (HR = 1.80, 95% CI, 1.53-2.11) by a fixed-effects model (P = 0.950, I2 = 0.0%), but we did not discover any significant outcome in studies of ovarian cancer or breast cancer (Figure 3C).  Similar to OS analyses, we also performed subtotal investigation for RFS analyses (Table 3). When stratified by dominant ethnicity, no statistically significant association between elevated miR-221 levels and RFS was observed in both Caucasian and Asian populations (Figure 2B). In subtotal analyses of main pathological type, the pooled outcome of adenocarcinoma subgroup did not reveal increased expression of miR-221 could significantly predict a poor RFS (Figure 3D). When stratified by the category of detected samples, the outcome of tissue subgroup was not statistically significant for the relationship of increased expression of miR-221 and RFS (Figure 3E). Finally, stratified by the category of malignant diseases, elevated expression of miR-221 exhibited a significant association with poorer RFS (HR = 2.43, 95% CI, 1.24-4.77) in HCC by a random-effects model (P = 0.091, I2 = 58.3%). In contrast, the pooled outcome in prostate cancer subgroup showed elevated miR-221 expression was significantly associated with a better RFS (HR = 0.51, 95% CI, 0.32-0.81) by a fixed-effects model (P = 0.831; I2=0.0%) (Figure 3F). | Results |
| **DISCUSSION** | | |  |
| Summary of evidence | 24 | Summarize the main findings including the strength of evidence for each main outcome; consider their relevance to key groups (e.g., healthcare providers, users, and policy makers). | Discussion |
| Limitations | 25 | Discuss limitations at study and outcome level (e.g., risk of bias), and at review-level (e.g., incomplete retrieval of identified research, reporting bias). | Discussion |
| Conclusions | 26 | In summary, we conclude that miR-221 is suitable to predict tumor prognosis in Asian populations, and is an ideal prognostic biomarker for HCC patients. Besides, elevated miR-221 expression in serum/ plasma samples is more convincing to predict a poor prognosis than detection of miR-221 in tissue samples. Detection of miR-221 in human peripheral blood samples possesses the advantages of low cost, convenience, and noninvasion, resulting in an effective method in monitoring cancer progression as well as assessing therapeutic efficacy in future. Considering the paucity of relevant data, further investigation and more studies are needed to focus on the relationship between the expression of miR-221 and cancer prognosis. | Conclusions |
| **FUNDING** | | |  |
| Funding | 27 | Describe sources of funding for the systematic review and other support (e.g., supply of data); role of funders for the systematic review. | n/a |

*From:*  Moher D, Liberati A, Tetzlaff J, Altman DG, The PRISMA Group (2009). Preferred Reporting Items for Systematic Reviews and Meta-Analyses: The PRISMA Statement. PLoS Med 6(6): e1000097. doi:10.1371/journal.pmed1000097

For more information, visit: **www.prisma-statement.org**.

Page 2 of 2
