# Supplementary material for: Prognostic Role of MicroRNA-221 in Various Human Malignant Neoplasms: A Meta-Analysis of 20 Related Studies
Source: PLoS One. 2014 Jan 27;9(1):e87606. doi: 10.1371/journal.pone.0087606 (PMC3903772; doi:10.1371/journal.pone.0087606)
Supplement: File S1 — PRISMA flow diagram. (DOC) [file pone.0087606.s002.doc]

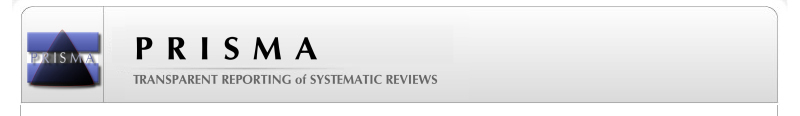
**PRISMA 2009 Flow Diagram**

**Screening**

**Included**

**Eligibility**

**Identification**

Records identified through database searching
(n = 532 )

Additional records identified through other sources
(n = 29 )

Records after duplicates removed
(n = 556 )

Records screened
(n = 556 )

Records excluded
(n = 483 )

Full-text articles assessed for eligibility
(n = 73 )

Full-text articles excluded, with reasons
(n = 53 )

Studies included in qualitative synthesis
(n = 20 )

Studies included in quantitative synthesis (meta-analysis)
(n = 20 )
